# Resting-state brain networks in neonatal hypoxic-ischemic brain damage: a functional near-infrared spectroscopy study

**DOI:** 10.1117/1.NPh.8.2.025007

**Published:** 2021-05-14

**Authors:** Shen Zhang, Cheng Peng, Yang Yang, Daifa Wang, Xinlin Hou, Deyu Li

**Affiliations:** a Beihang University, School of Biological Science and Medical Engineering, Beijing, China; b Peking University First Hospital, Department of Neonatal Ward, Beijing, China; c Beihang University, Advanced Innovation Center for Biomedical Engineering, Beijing, China; d Beihang University, State Key Laboratory of Software Development Environment, Beijing, China; e Beihang University, State Key Laboratory of Virtual Reality Technology and System, Beijing, China

**Keywords:** infants, hypoxic-ischemic brain damage, functional near-infrared spectroscopy, brain network, resting state

## Abstract

**Significance:** There is an emerging need for convenient and continuous bedside monitoring of full-term newborns with hypoxic-ischemic brain damage (HIBD) to determine whether early intervention is required. Functional near-infrared spectroscopy (fNIRS)-based resting-state brain network analysis, which could provide an effective evaluation method, remains to be extensively studied.

**Aim:** Our study aims to verify the feasibility of fNIRS-based resting-state brain networks for evaluating brain function in infants with HIBD to provide a new and effective means for clinical research in neonatal HIBD.

**Approach:** Thirteen neonates with HIBD were scanned using fNIRS in the resting state. The brain network properties were explored to attempt to extract effective features as recognition indicators.

**Results:** Compared with healthy controls, newborns with HIBD showed decreased brain functional connectivity. Specifically, there were severe losses of long-range functional connectivity of the contralateral parietal-temporal lobe, contralateral parietal-frontal lobe, and contralateral parietal lobe. The node degree showed a widespread decrease in the left frontal middle gyrus, left superior frontal gyrus dorsal, and right central posterior gyrus. However, newborns with HIBD showed a significantly higher local network efficiency (*p<0.05). Subsequently, network indicators based on small-worldness, local efficiency, modularity, and normalized clustering coefficient were extracted for HIBD identification with the accuracy observed as 79.17%.

**Conclusions:** Our findings indicate that fNIRS-based resting-state brain network analysis could support early HIBD diagnosis.

## 1 Introduction

Hypoxic-ischemic brain damage (HIBD) is among the leading causes of neonatal death and neurological disorders.^1^ Persistent brain injury in the neonatal period has been suggested to disrupt key structural development, which results in serious consequences such as white matter abnormalities, neuronal necrosis, and intracerebral hemorrhage. Nearly 25% of survivors present neurological-related sequelae, including mental retardation, paralysis, epilepsy, and other diseases.^2^^–^^4^ Typical neurological symptoms of HIBD deteriorate within a few days after birth; therefore, continuous monitoring and effective evaluation of brain function in these children could help determine whether targeted intervention is necessary and allow for decisive disease diagnosis and treatment.^5^

Currently, the clinical HIBD diagnosis mainly relies on two aspects. These include clinical characterization, which specifically refers to abnormal changes in consciousness, original reflection (there are some congenital reflexes in newborns, which reflect whether the body and nervous system function of the newborn is normal), and muscle tension,^6^ as well as detection of HIBD-induced lesions using ultrasound, computed tomography (CT), magnetic resonance imaging (MRI), and other medical imaging technologies. These classical technologies have their own advantages and limitations. Ultrasound has gradually optimized resolution in brain structure scanning, but it is insufficient at monitoring capabilities of functional hemodynamics. CT involves a certain radiation degree, with immature brain tissue having an unideal tolerance. MRI has a strong spatial resolution, which can accurately distinguish the perfusion level of regional cerebral blood flow. However, there is an emerging need for convenient and continuous bedside monitoring of neonates who are unable to undergo MRI due to clinical instability and/or the medical equipment required for therapeutic interventions. It would be a positive effort to satisfy the need by functional near-infrared spectroscopy (fNIRS) resting-state brain network analysis.

fNIRS is a relatively new non-invasive brain imaging technology and has attracted great attention from brain researchers due to its friendliness to the participants.^7^^,^^8^ More importantly, the main advantage of fNIRS in the diagnosis of HIBD is to support portable and continuous bedside monitoring. fNIRS allows us to obtain neonatal high-quality data sets within a few minutes. Notably, the data can be collected with the infants in a quiet or sleep state without the need to perform tasks or other auxiliary reagents (tranquilizers). The short preparation and detection period at the bedside means that pediatricians can record data repeatedly at any critical point. In addition, fNIRS avoids the effects of radiation on newborns compared with CT or positron emission CT.

Brain network analysis has been widely used in the evaluation of brain function. The human brain is a highly complex network system with numerous local or global topological features.^9^^,^^10^ Some synchronous low-frequency fluctuations are associated with neural activity between some brain regions in the resting state, which indicates that organized activities between different brain regions contribute to maintaining the mechanism of brain activity.^11^^,^^12^ Different from a random network, the brain functional network is economical, which ensures that the brain can differentiate and integrate information efficiently, providing the physiological basis for information processing and mental representations.^13^ Bullmore and Sporns^14^ believe that brain networks can be examined by critical properties of graph theory, such as clustering coefficient, node degree, efficiency, and modularity. These metrics of graph theory provide key information about the network structure and describe the specific organizational style of the network. Over the past decade, resting-state brain networks have had great utility in brain function assessment, especially when assessing neurocognitive development in newborns. Relevant fMRI studies have shown some basic functional networks in healthy newborns;^15^ moreover, the precursors of some advanced networks have been identified. Studies have demonstrated the presence of the default mode network of the primary motor cortex and sensory cortex.^16^ Smyser et al.^17^ used fMRI to explore the resting-state connectivity of premature infants. The results showed that the most obvious decrease of functional connectivity was in the area near the injured site. They also confirmed that abnormal development of periventricular white matter would lead to a decrease of network connectivity. Tusor^18^ studied 15 infants with HIBD, and conventional MRI showed that there were varying degrees of damage to white matter and gray matter in the cohort. In these infants, typical resting-state networks, including auditory, somatomotor, visual, and default pattern networks, were identified. In addition, the long-distance connection of the unilateral brain in children with HIBD was weakened.

A series of advances has been made in the study of the neonatal fNIRS connectivity. Homae et al.^19^ conducted follow-up fNIRS assessments of healthy newborns for 6 months and observed gradual complication and enhancement of the functional network in each region of the neonatal cerebral cortex. Makiko et al.^20^ found that infants with Down’s syndrome had lower connectivity and different local hemodynamics, which demonstrated the potential of fNIRS for clinical use in infants. Lin et al.^21^ utilized resting-state fNIRS imaging data to explore topological changes in network organization during development from early childhood and early adolescence to adulthood, and the results showed the developmental maturity of important functional brain organization in early childhood. Kelsey et al.^22^ explored the link between gut microbiome, brain, and behavior in 63 newborn infants by resting-state fNIRS. They found that the composition of gut microbiota is related to the individual differences of brain network connectivity, which in turn mediates the individual differences of infant behavior temperament. Their findings indicate that the gut microbiome plays an important role in human development.

However, as far as we know, the existing studies on the functional connectivity of the neonatal brain mostly focus on preterm infants (gestational age < 37 weeks) and do not deeply determine the changes of their network properties. Full-term infants with HIBD often miss the treatment window because of their atypical clinical symptoms, until serious complications such as hydrocephalus are found. The specific network properties have not been extracted as sensitive biological factors for early auxiliary diagnosis of HIBD. Given the aforementioned findings, this study aims to use fNIRS to record the resting-state data of full-term infants with HIBD for constructing a functional network that covers the prefrontal, parietal, and temporal lobes, to observe the local or global topological features of HIBD using network analysis, and to extract sensitive biological factors to achieve effective recognition of HIBD.

## 2 Methods

In this experiment, fNIRS was applied to record resting-state signals from neonates with HIBD. We constructed a whole-brain functional network based on the between-channel correlation of the sequences of hemoglobin concentration. Functional connections between six early-developing regions of interest (ROIs) were explored. Using comparisons, sensitive network indicators were extracted as features and used to input support vector machines (SVM) for training and testing. This study was conducted according to the Declaration of Helsinki and approved by the local Ethics Committee of Beihang University.

### 2.1 Participants

This study enrolled participants from the pediatric neonatal ward of the Peking University First Hospital. The inclusion criteria were as follows: (1) term neonates with a gestational age of 37 to 44 weeks; (2) having a HIBD diagnosis (mainly including diffuse white matter abnormalities, periventricular leukomalacia, blood oxygen ischemic encephalopathy, intraventricular hemorrhage, hemorrhagic ventricular dilatation, hemorrhagic hydrocephalus, and periventricular hemorrhagic infarction); and (3) having consent from the legal guardian. All relevant assessments were obtained within 72 h of the baby’s birth since typical neurological symptoms of HIBD appear within 6 to 12 h after birth and peak at 72 h. The acquisition time was strictly controlled to avoid the effect of our clinical intervention. All infants were monitored after full lactation and natural sleep. Two infants woke up and cried, so they could not complete the monitoring. They were excluded in the follow-up analysis. After early screening and subsequent visits, 13 eligible newborns with HIBD and 13 healthy newborns as controls were enrolled in the experiment. The socio-demographic information for this study is shown in Table 1.

**Table 1:** Socio-demographic information for this study.

Infant no.	1	2	3	4	5	6	7	8	9	10	11	12	13
HIBD													
Gender	M	M	M	F	M	M	F	F	M	F	M	M	M
GA (week)	38	37	39	41	39	40	39	37	40	39	38	38	39
Birth weight (kg)	2.81	2.46	3.60	3.05	2.60	2.68	3.70	2.15	3.80	3.60	3.71	3.60	2.72
Delivery	C	T	T	T	T	T	C	T	T	T	C	T	C
Feeding	Mix	Milk	Mix	Mix	Mix	Mix	Mix	Mix	Mix	Mix	Mix	Mix	Mix
Healthy control													
Gender	F	M	M	M	M	F	M	F	F	M	F	F	M
GA (week)	39	38	40	38	40	39	38	39	40	39	40	38	39
Birth weight (kg)	3.28	3.07	3.26	2.78	3.48	3.04	3.19	3.31	3.75	3.26	2.58	2.92	3.01
Delivery	T	C	T	C	C	C	C	T	T	T	C	C	C
Feeding	Mix	Mix	Mix	Mix	Milk	Mix	Mix	Mix	Mix	Mix	Milk	Mix	Milk

Note: GA = gestational age; M = male; F = female; C = cesarean delivery; T = transvaginal delivery; mix = breast feeding + formula milk powder.

### 2.2 Data Acquisition

The fNIRS signals were acquired using a multichannel fNIRS system (NirSmart-2416, HuiChuang, China) with two wavelengths (760 and 850 nm) at a sampling rate of 10 Hz.

**Fig. 1:**
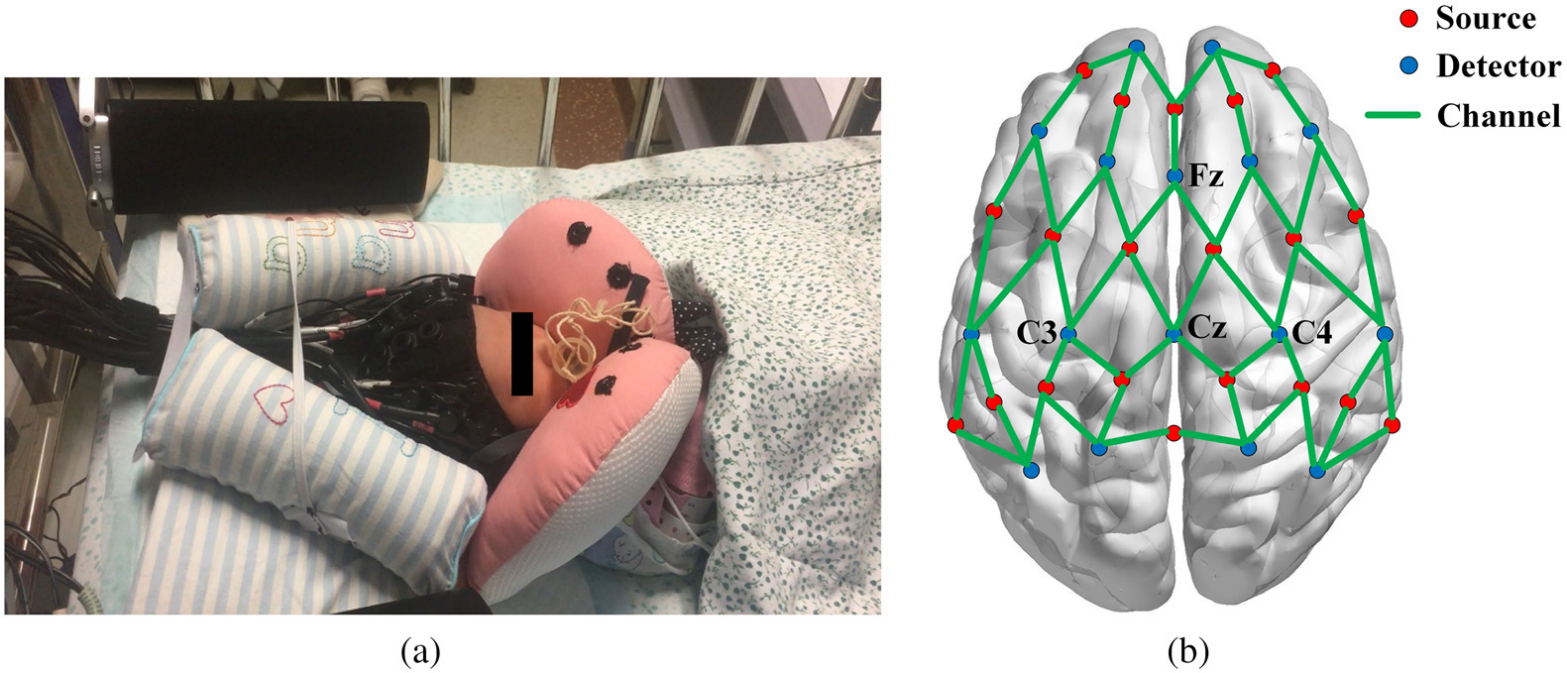
(a) Experimental settings. (b) Schematic illustration of the fNIRS layout (45 channels, 20 sources, and 16 detectors). The green lines represent channels, and the nodes represent optical probes. The arrangement covers the prefrontal, temporal, and parietal lobes.

An experimental platform dedicated to newborns was established [see Fig. 1(a)]. To reduce interference from the external environment, the newborns were tested in a room with dim light and sound insulation effects. Before being tested, the newborn was placed in a supine position in the crib and the head was fixed using measuring aids. Subsequently, the special head cap covered the early developing primary functional areas, including the prefrontal, temporal, and parietal lobes. As shown in Fig. 1(b), the cap was formed by 20 sources and 16 detectors, which comprised the channels. To ensure the safety and comfort of the newborns, the probes and head cap were made of soft materials to achieve soft contact with the scalp. Table 2 shows the specific correspondence between the channels and brain regions. Resting-state data were collected for 10 min to subsequently construct brain functional networks, following Wang et al.^23^ who found that the functional connectivity remained stable only when the fNIRS data acquisition duration was longer than 7 min. NirSmart-2416 supported the automatic adjustment of the source power and detector gain to optimize signal quality. The average signal-to-noise ratio of channels used was 22.2±12.1  dB.

**Table 2:** The MNI coordinates and anatomical labels corresponding to the measurement channels.

Channel		Length of the channel (mm)	MNI coordinates			ROI	Anatomic label
			x	y	z		
1	TP7-T7	20	−45	−25	−2	LTL	Middle temporal gyrus left
2	TP7-P7	25	−41	−42	−1	LTL	Middle temporal gyrus left
3	FT7-T7	20	−45	−8	−2	LTL	Temporal pole (superior) left
4	FT7-F7	20	−41	10	−3	LTL	Superior temporal gyrus left
5	FC5-T7	25	−46	−7	8	LTL	Heschl gyrus left
6	FC5-F7	25	−42	11	7	LTL	Inferior frontal gyrus (opercular) left
7	FC5-C3	30	−44	−6	31	LPL	Postcentralgyrus left
8	FC5-F3	25	−41	14	26	LPL	Middle frontal gyrus left
9	CP5-P7	25	−43	−44	10	LTL	Middle temporal gyrus left
10	CP5-C3	30	−47	−27	32	LPL	Inferior parietal lobule left
11	CP5-P3	25	−42	−48	30	LPL	Angular gyrus left
12	AF7-F7	20	−37	26	−2	LPFC	Inferior frontal gyrus (triangular) left
13	AF7-Fp1	25	−26	39	−2	LPFC	Middle frontal gyrus left
14	AF3-F3	25	−29	34	25	LPFC	Middle frontal gyrus left
15	AF3-Fp1	25	−17	44	10	LPFC	Superior frontal gyrus (dorsal) left
16	CP1-C3	30	−35	−27	55	LPL	Postcentralgyrus left
17	CP1-P3	30	−31	−50	47	LPL	Superior parietal gyrus left
18	FC1-C3	30	−33	−5	50	LPL	Precentralgyrus left
19	FC1-F3	30	−30	17	42	LPL	Middle frontal gyrus left
20	FC1-Fz	30	−14	17	53	LPL	Superior frontal gyrus (dorsal) left
21	FC1-Cz	30	−15	−4	61	LPL	Supplementary motor area left
22	AFz-Fp1	30	−10	45	13	LPFC	Superior frontal gyrus (medial) left
23	AFz-Fz	25	−2	36	37	Prefrontal cortex	Superior frontal gyrus (medial)
24	AFz-Fp2	30	7	45	14	RPFC	Superior frontal gyrus (medial) right
25	AF4-Fp2	25	14	43	11	RPFC	Superior frontal gyrus (dorsal) right
26	AF4-F4	25	25	32	28	RPFC	Middle frontal gyrus right
27	FC2-Fz	30	6	18	53	RPL	Superior frontal gyrus (dorsal) right
28	FC2-Cz	30	7	−3	61	RPL	Supplementary motor area right
29	FC2-F4	30	24	16	45	RPL	Middle frontal gyrus right
30	FC2-C4	30	27	−4	52	RPL	Middle frontal gyrus right
31	AF8-Fp2	25	23	38	0	RPFC	Middle frontal gyrus right
32	AF8-F8	20	32	25	0	RPFC	Inferior frontal gyrus (triangular) right
33	CP2-C4	30	28	−26	56	RPL	Postcentralgyrus right
34	CP2-P4	30	26	−50	50	RPL	Superior parietal gyrus right
35	FC6-F4	25	35	13	30	RPL	Middle frontal gyrus right
36	FC6-F8	25	37	10	11	RTL	Inferior frontal gyrus (opercular) right
37	FC6-C4	30	39	−5	34	RPL	Postcentralgyrus right
38	FC6-T8	25	41	−6	11	RTL	Heschl gyrus right
39	FT8-F8	20	36	8	0	RTL	Superior temporal gyrus right
40	FT8-T8	20	40	−7	0	RTL	Temporal pole (superior) right
41	CP6-C4	30	41	−26	37	RPL	Inferior parietal lobule right
42	CP6-P4	25	37	−46	34	RPL	Angular gyrus right
43	CP6-P8	25	40	−43	14	RTL	Middle temporal gyrus right
44	TP8-T8	20	41	−24	1	RTL	Middle temporal gyrus right
45	TP8-P8	25	38	−41	2	RTL	Middle temporal gyrus right

### 2.3 Data Preprocessing

The NirSpark software (HuiChuang, China) package was used to preprocess fNIRS signals. During fNIRS scans, the newborns occasionally had involuntary sudden head movements (even in sleep). Motion artifacts affect functional connectivity analysis deeply and have attracted much attention. The commonly used correcting approaches include spline interpolation, wavelet analysis, principal component analysis, Kalman filtering, etc. Each method has its own advantages. The positive aspect of spline interpolation is that it only corrects the pre-localized artifacts without modifying the other portions of the time series. Therefore, the spline interpolation method was used to amend motion artifacts, which were manifested as an impulse or cliff-type jumps caused by the relative sliding of the scalp and probes.^24^^–^^26^ Subsequently, 0.01- to 0.1-Hz bandpass filtering was performed to remove the noise based on physiological fluctuations such as pulse and respiration.^27^ Then the modified Beer–Lambert law was used to transform light intensity data into the relative change of the concentration of oxygenated (HbO) and deoxygenated hemoglobin (HbR) as follows:

$$\Delta {\mathrm{OD}}^{\lambda_{i}}=(\varepsilon_{\mathrm{Hbo}}^{\lambda_{i}}\Delta C_{\mathrm{Hbo}}+\varepsilon_{\mathrm{HbR}}^{\lambda_{i}}\Delta C_{\mathrm{HbR}})\times r\times {\mathrm{DPF}}^{\lambda_{i}},\;i=1,2,$$ (1)

where the variable ε is the wavelength-dependent extinction coefficient for each hemoglobin type. The change in light absorption, which is referred to as the delta optical density, is represented as ΔOD. $\Delta C_{\mathrm{HbO}}$ and $\Delta C_{\mathrm{HbR}}$ represent the relative concentration changes of HbO and HbR, respectively. The DPF (differential path-length factor) accounts for the true effective path length between the source and detector, while r represents the linear distance between the paired probes. The DPF is related to the wavelength of the incident light and the distance between sources and detectors. Based on a related study by van der Zee et al.,^28^ DPF=4 was considered appropriate for this study.

### 2.4 Brain Functional Networks Construction

During the past 20 years, brain studies have increasingly applied EEG-, fMRI-, and fNIRS-based brain network analysis, as well as other brain imaging techniques. Studies have shown that graph theory-based brain network theory is an effective tool for analyzing brain structure and function, which reveals numerous potential operating mechanisms and features.^29^

During advanced cognitive processing, there is cooperation among brain regions with a consistent hemoglobin supply. The transformed HbO or HbR sequences were used to construct and evaluate the correlation between 45 channels through Pearson’s correlation coefficient as follows:

$$r=\frac{\Sigma_{i=1}^{n}(X_{i}-\overset{¯}{X})(Y_{i}-\overset{¯}{Y})}{\sqrt{\Sigma_{i=1}^{n}{\left(X_{i}-\overset{¯}{X}\right)}^{2}}\sqrt{\Sigma_{i=1}^{n}{\left(Y_{i}-\overset{¯}{Y}\right)}^{2}}},$$ (2)

where X and Y represent the time series of hemoglobin concentration in the different channels or ROIs, respectively, and r is the correlation coefficient. Thus, a 45×45 functional connectivity matrix could be obtained from each participant. Subsequently, Fisher’s r-to-z transformation was applied to convert these correlation coefficients to z-scores for improved normality.

Forty-five channels were divided into six ROIs based on their location (see Table 1), including the left prefrontal cortex (LPFC), left temporal lobe (LTL), left parietal lobe (LPL), right prefrontal cortex (RPFC), right temporal lobe (RTL), and right parietal lobe (RPL). Moreover, we averaged the time series of all channels in each region and calculated the between-region r to evaluate the between-ROI correlation.

Using threshold sparsity, the correlation matrix was transformed into a binary matrix followed by the construction of the specific brain network model of HIBD. The thresholds were selected to ensure network integrity and small-world attributes. The sparsity parameter was selected to determine the ratio of the number of existing edges to the maximum possible edges, which has a great impact on the topology of the network. Usually, researchers apply multiple thresholds and analyze the topological properties of brain networks. Bassett et al.^30^ used a sparsity range (5%<s<25%, stepsize=1%) to explore the network properties. Lin et al.^21^ and Wang et al.^23^ investigated the relationship between function connectivity and fNIRS data length with 10%<s<50%. This is because the real difference of topological attributes between states is likely to cover a sparsity interval (called sparsity segment), rather than a few sporadic sparsity levels. Random noise only has a very low probability to form a statistically significant sparsity segment. In this study, 36 brain network models were constructed to assess HIBD characteristics at different scales (5%<s<40%, stepsize=1%). Further, we generated random networks with the same number of nodes, number of edges, and degree distribution as the actual network to verify the reliability of the real network. Several common global network and regional node metrics were used to evaluate global and local topological features, including clustering coefficient ($C_{p}$), small-worldness (σ), modularity (Q), local efficiency ($E_{\mathrm{loc}}$), and global efficiency ($E_{\mathrm{glob}}$). The calculation formula is as follows:^11^^,^^31^

$$C_{P}=\frac{1}{N}\underset{i\in G}{\sum }\frac{E_{i}}{D_{i}(D_{i}-1)/2},$$ (3)

where N represents the number of nodes in the unweighted network G, $D_{i}$ is the number of edges connected to the i’th node, and $E_{i}$ is the number of edges in the subgraph. The clustering coefficient reflects the local interconnectivity of a network,

$$\sigma =\frac{C_{p}\_\gamma }{L_{p}\_\lambda }.$$ (4)

The characteristic path length $L_{p}$ of graph G is defined as the average of the shortest path lengths between all node pairs in network G. Specifically, $C_{p}\_\gamma =C_{p}^{\text{real}}/C_{p}^{\text{rand}}$, where $C_{p}^{\text{rand}}$ is the average value of the corresponding parameters derived from 1000 matched random networks with the same number of nodes, edges, and degree distribution as the real brain network. Similarly, $L_{p}\_\lambda =L_{p}^{\text{real}}/L_{p}^{\text{rand}}$ could be calculated to examine the small-world attributes of the networks,

$$Q(p)=\overset{m-1}{\underset{M}{\sum }}[\frac{l_{m}}{L}-{\left(\frac{d_{m}}{2L}\right)}^{2}],$$ (5)

where M is the number of modules, L is the total number of edges of the network, $l_{m}$ is the total number of edges in module m, and $d_{m}$ represents the sum of the degrees of the nodes in module m. Modularity is defined as the largest value of modularity measures associated with all possible configurations of modules,

$$E_{\mathrm{glob}}=\frac{1}{N(N-1)}\sum_{i\neq j\in G}\frac{1}{d_{ij}},$$ (6)

$$E_{\mathrm{loc}}=\frac{1}{N}\sum_{i\in G}E_{\mathrm{glob}}(i),$$ (7)

where $d_{ij}$ is the shortest distance between node i and node j and N is the number of nodes of G. Concretely, $E_{\mathrm{glob}}$ delivers the efficiency of parallel information transfer in the network, while $E_{\mathrm{loc}}$ measures the local efficiency of information transfer in the immediate neighborhood of each node. Similar to the normalized clustering coefficient, the normalized local and global efficiency were also calculated as follows: $E_{\mathrm{loc}}\_\gamma =E_{\mathrm{loc}}^{\text{real}}/E_{\mathrm{loc}}^{\text{rand}}$ and $E_{\mathrm{glob}}\_\gamma =E_{\mathrm{glob}}^{\text{real}}/E_{\mathrm{glob}}^{\text{rand}}$, respectively. More concretely, $E_{\mathrm{loc}}^{\text{rand}}$ and $E_{\mathrm{glob}}^{\text{rand}}$ were the average value of the corresponding parameters derived from 1000 matched random networks with the same number of nodes, edges, and degree distribution as the real brain network.

### 2.5 Statistical Analysis

Two-sample t-tests and false discovery rate (FDR) correction were applied to compare the differences between the HIBD and healthy control (HC) groups. To examine the small-world attributes of HIBD networks, we showed the small-worldness, local efficiency, modularity, and normalized clustering coefficient at a threshold of 0.3, and the statistical difference between HIBD and HC was exhibited.

Based on differences in brain networks, we extracted the most recognizable small-world attributes and operational efficiency as the feature input to the SVM for model training, which could provide concise and efficient auxiliary indicators for HIBD clinical diagnosis.

The small-worldness, local efficiency, modularity, and normalized clustering coefficient at a threshold in the range of 0.3 to 0.34 were selected as features. Thus, 4×5=20 features could be extracted from each sample. We randomly divided the 13 HIBD samples, as well as the HC samples, into the training group and test group (10:3). Then the SVM was conducted for the binary classifications of the HIBD and HC. This study selected a linear kernel function and fivefold cross-validation mode.^32^^,^^33^ Cross folding for internal validity and grid search methods were used to identify the optimal parameters c and g. In addition, the receiver operating characteristic (ROC) curve approach was applied to evaluate the sensitivity and specificity of the four types of significant differential features. The area under the ROC curve (AUC) was conducted to quantify the performances of these features in detecting HIBD.

## 3 Results

We explored the structural characteristics of the resting brain network in infants with HIBD and assessed for differences in the network phenomenon compared with healthy newborns. In this study, we mainly used HbR signals to characterize the topological development of functional brain networks since they are generally more reliable for most brain network metrics.

### 3.1 Channel-Based Functional Connectivity

**Fig. 2:**
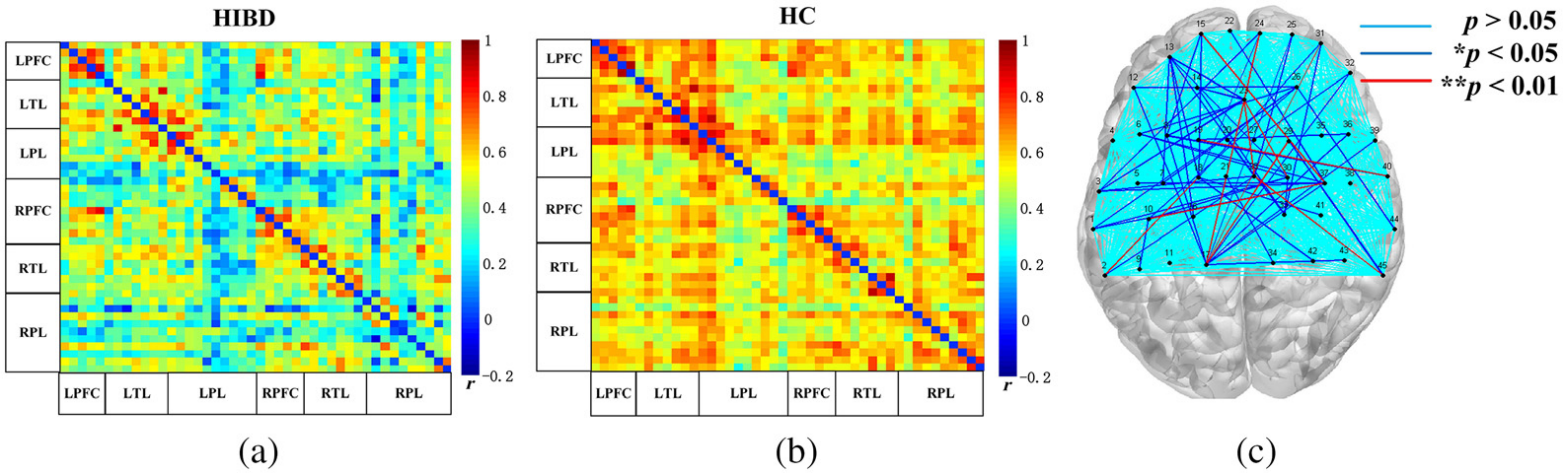
Grand-averaged correlation matrix: (a) infants with HIBD and (b) healthy infants. Axes represent the regions. Each channel with its correlation coefficient set at zero (the diagonal line). LPFC, left prefrontal cortex; LTL, left temporal lobe; LPL, left parietal lobe; RPFC, right prefrontal cortex; RTL, right temporal lobe; RPL, right parietal lobe. (c) The inter-group differences in actual channels. The dark blue lines represent connections with significant differences (*p<0.05) and the red lines represent connections with extremely significant difference (**p<0.01).

The grand-average correlation coefficient matrices of infants with HIBD and healthy infants are presented in Figs. 2(a) and 2(b), respectively, and were used to describe the between-channel correlation of the whole brain in each group. Compared with the control group, the HIBD group has significantly weakened functional connections (mean±SD: 0.57±0.10 and 0.41±0.14, respectively). We used two-sample, two-sided power analysis to calculate the statistical power. The sample size of each group was 13, and the overall standard deviation was 0.14. The final calculated power was 83.06% at the significance level of 5%. For the results of Fig. 2(c), there were 62 connections with a significant difference (*p<0.05) and 9 connections with a significant difference (**p<0.01). This indicates that HIBD caused diffuse functional decline throughout the brain.

### 3.2 ROI-Based Functional Connectivity

**Fig. 3:**
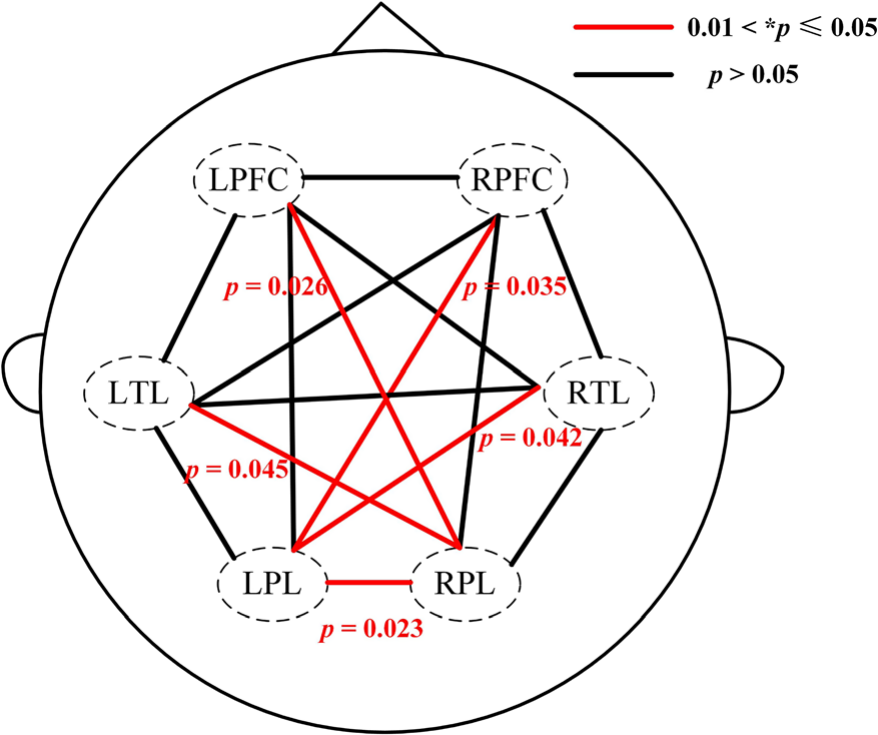
The p values of the inter-group t-test of functional connections between all ROIs. The dashed circles represent six ROIs. Red lines indicate significant inter-group differences in the regional connection (0.01<*p≤0.05).

To further explore the between-ROI connectivity characteristics, the time series of six ROIs’ internal channels were averaged, and two-sample t-tests and FDR correction were applied to compare the differences between the HIBD and HC groups. Compared with the control group, the HIBD group had significantly lower cross-interval brain functional connectivity intensity in LTL-RPL [t(24)=−2.08, *p=0.045], LPFC-RPL [t(24)=−2.34, *p=0.026], LPL-RTL [t(24)=−2.11, *p=0.042], LPL-RPFC [t(24)=−2.21, *p=0.035], and LPL-RPL [t(24)=−2.38, *p=0.023], as shown in Fig. 3. There was a greatly significant difference in long-distance connectivity associated with the parietal lobe.

### 3.3 Functional Networks

Based on brain functional connectivity, we constructed HIBD brain network models at different scales using threshold sparsity. Given the underlying physiological mechanisms, delayed brain development caused by hypoxia-ischemia probably affected the formation of important cortical hubs and reduced the efficiency of interregional cooperation. Therefore, we explored the brain network attributes of infants with HIBD from the central point, network efficiency, and modularity. Moreover, we attempted to elucidate the internal basis for clinical disease characterization.

**Fig. 4:**
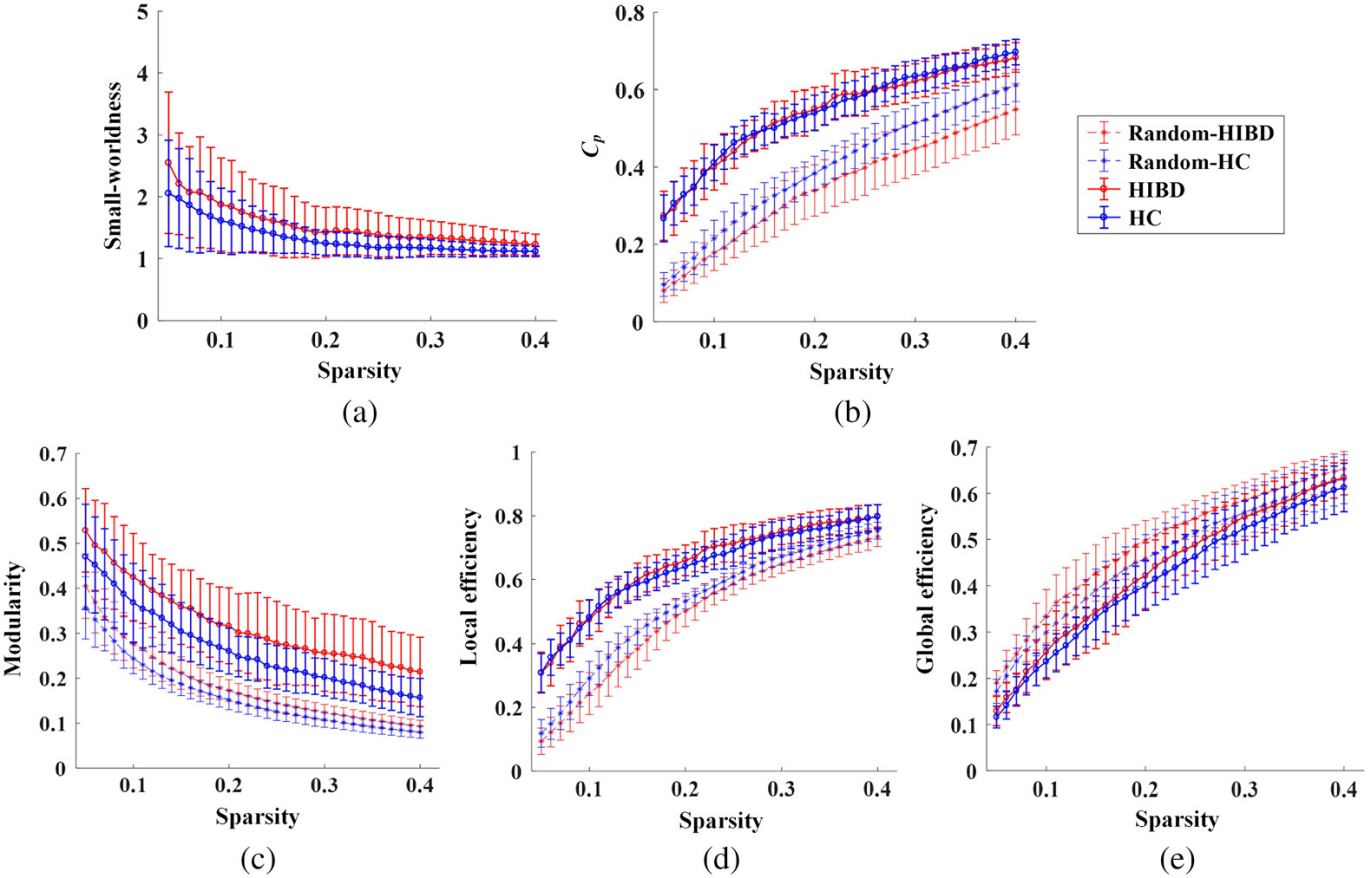
The functional network metrics in the range of the sparsity thresholds (0.05 to 0.4). (a) The small-worldness, (b) the normalized clustering coefficient, (c) the modularity, (d) the local efficiency, and (e) the global efficiency. Red and blue curves with circles represent the HIBD and healthy control groups, respectively. The curves with an asterisk represent the mean and error bars of the matched random networks.

Figure 4 demonstrates the functional network metrics of real (solid line) and random (dashed line) networks with an increasing threshold (0.05 to 0.4). It can be seen from Fig. 4(a) that the small-worldness of the HIBD and HC groups were both larger than 1 at all thresholds, which indicates that the brain networks in newborns have small-world attributes. The $C_{p}$, modularity, and local efficiency of brain networks were large than those of the random networks [see Figs. 4(b)–4(d)], whereas the global efficiency values of the networks were slightly lower than those of the matched random networks [Fig. 4(e)]. Compared with the HC group, the HIBD group showed higher modularity and small-worldness values, whereas the clustering coefficient and network efficiency of the two groups were comparable.

**Fig. 5:**
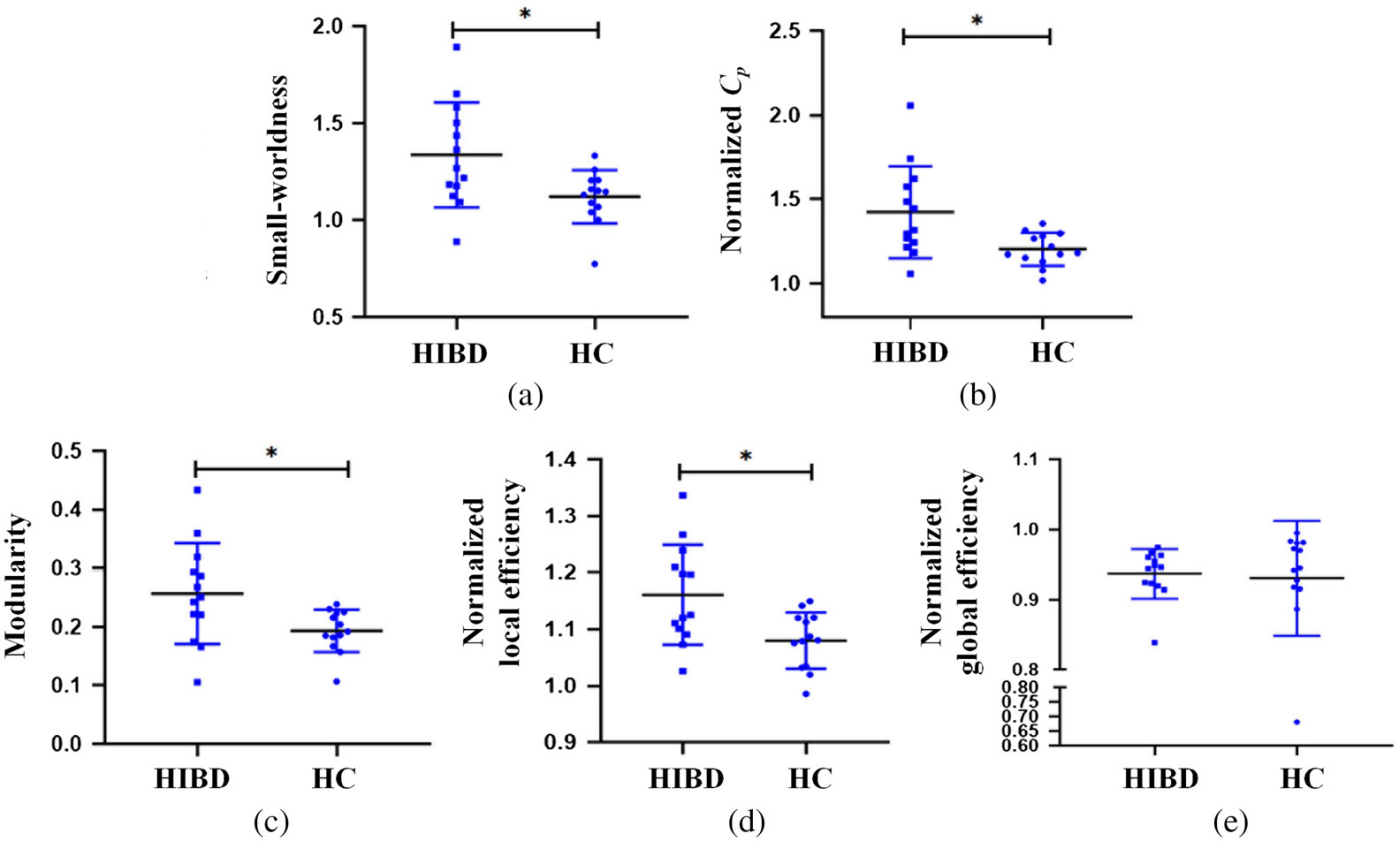
The group differences in the global network metrics with the sparsity threshold at 0.3. (a) The small-worldness, (b) the normalized clustering coefficient, (c) the modularity, (d) the normalized local efficiency, and (e) the normalized global efficiency. Asterisk indicates a significant difference (*p<0.05).

The group differences in the global network metrics with the sparsity threshold at 0.3 are shown in Fig. 5. The results demonstrated that the small-worldness [HIBD: 1.31±0.23; HC: 1.14±0.12; t(24)=2.27, *p=0.037], normalized $C_{p}$ [HIBD: 1.42±0.26; HC: 1.24±0.11; t(24)=2.26, *p=0.039], modularity [HIBD: 0.26±0.08; HC: 0.20±0.04; t(24)=2.21, *p=0.043], and normalized local efficiency [HIBD: 1.16±0.09; HC: 1.10±0.05; t(24)=2.41, *p=0.024] of HIBD networks were significantly higher than those of the HC group, while the normalized global efficiency [HIBD: 0.94±0.04; HC: 0.93±0.08; t(24)=0.25, p=0.801] of the HIBD group and HC group had no statistical differences.

In addition, we expect to mine as many effective features as possible in a fixed threshold range for the needs of clinical diagnosis. According to the statistical results, 0.3 to 0.34 is a noticeable threshold range. Table 3 shows the details of the four kinds of effective features used for classification.

**Table 3:** Details of features with thresholds of 0.3 to 0.34 used for classification.

	Sparsity threshold	t	p	HIBD (Mean±SD)	HC (Mean±SD)
Small-worldness	0.30	2.27	0.037	1.31±0.23	1.14±0.12
	0.31	2.14	0.048	1.33±0.25	1.16±0.13
	0.32	2.22	0.042	1.32±0.24	1.15±0.12
	0.33	2.27	0.037	1.31±0.23	1.14±0.12
	0.34	2.17	0.046	1.29±0.23	1.14±0.11
Normalized Cp	0.30	2.26	0.039	1.42±0.26	1.24±0.11
	0.31	2.32	0.035	1.41±0.25	1.23±0.11
	0.32	2.42	0.029	1.40±0.24	1.22±0.10
	0.33	2.50	0.025	1.38±0.23	1.21±0.10
	0.34	2.46	0.027	1.36±0.22	1.20±0.10
Modularity	0.30	2.21	0.043	0.26±0.08	0.20±0.04
	0.31	2.42	0.028	0.25±0.08	0.19±0.04
	0.32	2.34	0.033	0.25±0.08	0.19±0.04
	0.33	2.46	0.026	0.25±0.08	0.18±0.04
	0.34	2.40	0.030	0.24±0.08	0.17±0.04
Normalized local efficiency	0.30	2.41	0.024	1.16±0.09	1.10±0.05
	0.31	2.39	0.025	1.15±0.08	1.09±0.05
	0.32	2.39	0.025	1.14±0.08	1.08±0.05
	0.33	2.68	0.013	1.14±0.07	1.08±0.05
	0.34	2.64	0.014	1.13±0.07	1.07±0.05

Note: p<0.05 indicates a significant difference.

**Fig. 6:**
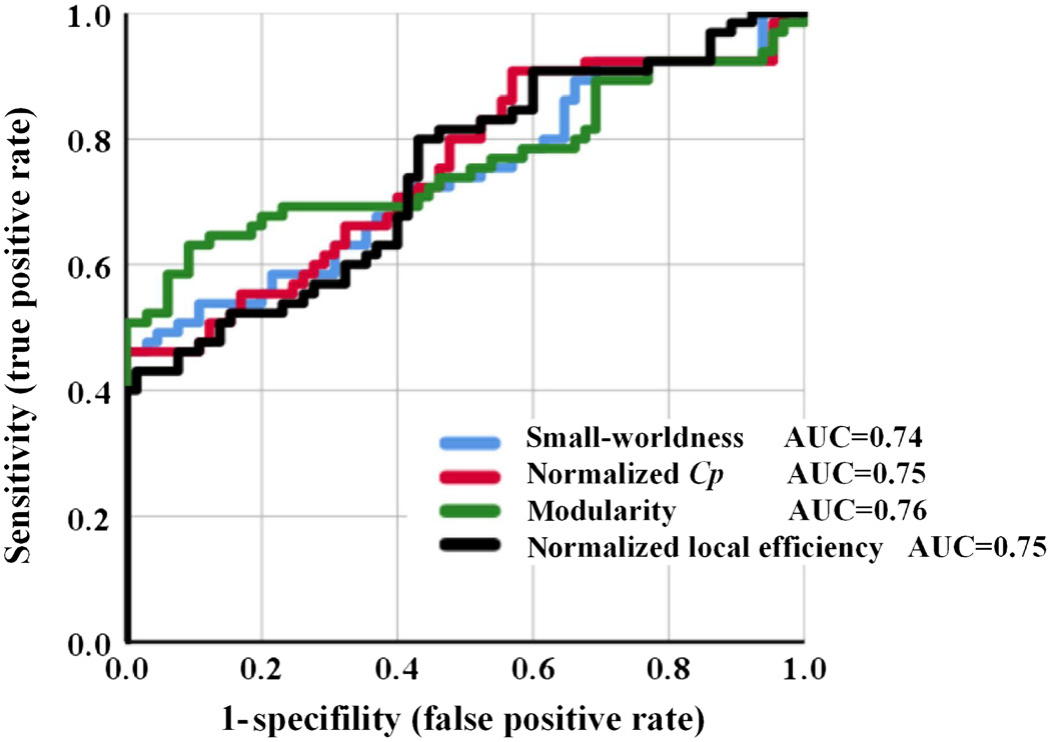
The ROC curves of small-worldness, normalized Cp, modularity, and normalized local efficiency, plotted in blue, red, green, and black, respectively. The AUC value for the four types of features is 0.74, 0.75, 0.76, and 0.75, correspondingly.

The AUC was conducted to quantify the performances of these features in detecting HIBD. The AUC value for small-worldness, normalized Cp, modularity, and normalized local efficiency is 0.74, 0.75, 0.76, and 0.75, respectively (see Fig. 6).

**Fig. 7:**
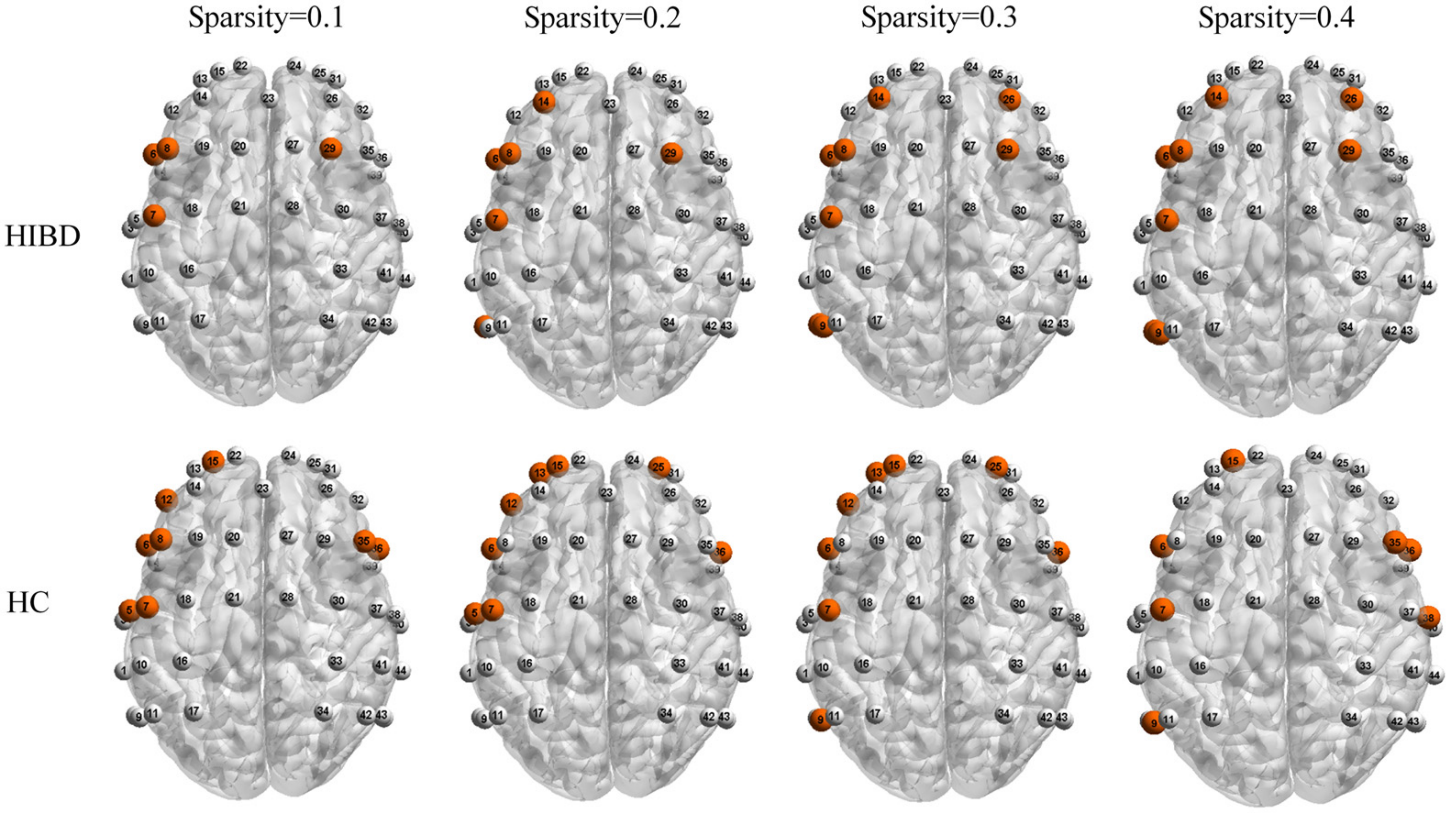
Grand-averaged central node of the two groups at four thresholds.

Figure 7 shows the grand-average central nodes of both groups, which reflected the key node distribution in the newborn brain. At four different sparsity scales, all neonates showed the central development characteristics of lateralization; specifically, there was a higher number of central nodes on the left side than on the right side, which was consistent with previous findings. Furthermore, the difference of distribution of the central nodes between groups was relatively large when the threshold is 0.1 and 0.2, but gradually disappears when the threshold was further increased.

### 3.4 Sensitive Feature Extraction

Based on the aforementioned network properties of infants with HIBD, we extracted the most recognizable small-world properties and operational efficiency as the feature input to the SVM for model training to determine efficient and convenient clinical evaluation for HIBD.

After analyzing the network characteristics of two data types, the four types of network indices, i.e., small-worldness, normalized local efficiency, modularity, and normalized clustering coefficient, all showed a higher sensitivity for identifying infants with HIBD at a threshold of 0.3 to 0.34. Therefore, we selected 4×5=20 network features of each sample. The optimal classification rate of the final model was 79.17%, and the mean classification accuracy was 72.92±4.53%. These findings regarding the ideal classification show that fNIRS analysis of the resting-state brain network can effectively detect functional abnormalities in children with HIBD and has clinical utility for early disease detection and intervention.

## 4 Discussion

For the channel-based results, HIBD significantly reduced resting-state brain functional connectivity across the whole brain in infants. The respective correlation matrix of the two data groups revealed that the HIBD group had significantly weaker brain functional network connectivity of infants than the control group. This is consistent with the characteristics of whole brain involvement in perinatal brain injury. In the early postnatal period, the brain of newborns develops rapidly under the joint stimulation of endogenous and environmental factors, and synaptic connections are reshaped under the joint influence of the body itself and the external environment. The findings of Fransson et al.^15^^,^^16^ suggest that basic functional networks already exist and rapidly develop in healthy newborns. Perinatal brain injury may be related to many factors such as abnormal synaptic development and decreased nerve cell transporters, which is also associated with dysgenesis of functional connectivity.

For the ROI-based results, there were severe losses of long-range functional connectivity between the brain regions of infants with HIBD. This phenomenon was observed between the contralateral parietal-temporal lobe, contralateral parietal-frontal lobe, and contralateral parietal lobe. This is consistent with previous findings regarding brain dysfunction in preterm infants. Tusor^18^ observed decreased resting-state single-side long-distance connections compared with healthy newborns. This suggests that when brain injury occurs, especially in the acute phase, bilateral parietal lobes may be most heavily involved. The involvement further weakens the long-distance information transmission, which limits the efficiency of synergistic cooperation between multiple brain regions. This is because, during brain development, HIBD-induced deficiency in blood oxygen supply results in vasoconstriction and white matter deletion; moreover, it may progress to involve neuronal necrosis of the overlying cortex. This hinders the synergistic cooperation between brain regions in newborns, which results in the infants presenting cognitive impairment with clumsiness or spasticity later in life.

The functional networks of infants with HIBD have a stronger ability of local information transmission. Analysis of small-world attributes and operational efficiency showed that the network normalized clustering coefficient, small world, normalized local efficiency, and modularity level were larger in the HIBD group than in the control group in the left auxiliary motor area, RTL area, and right parietal upper gyrus. This phenomenon was reported in an fMRI-based study on extreme preterm neonates compared with normal neonates. Elveda et al.^34^ found that the small-world degree of extremely premature infants was significantly larger than that of normal newborns at partial thresholds. This could be attributed to extremely premature infants having significantly enhanced intra-regional connectivity between the motor region and the auditory network region, which leads to the increased clustering coefficient in some regions and the increase of the small-world degree. This indicates that the functional connections of the infants also redistribute after acute brain injury, which is consistent with the redistribution of cerebral hemodynamics. The observed increase in local efficiency and the decline in overall ability could be attributed to the compensation effect. The less damaged functional area responsible for basic survival needs demonstrates the phenomenon of excessive compensation, which causes internal network overdevelopment. Contrastingly, severely damaged high-level functional hubs show impeded development. This phenomenon may be involved in the prognosis of patients with mental retardation, cerebral palsy, and other cognitive disorders.

Graph theory analysis based on functional connectivity can derive summary parameters to describe and quantify aberrant communication patterns associated with brain injury. After screening, network indicators based on small-worldness, local efficiency, modularity, and normalized clustering coefficient allowed for efficient HIBD identification. The AUC values demonstrated the ability of all of these features in HIBD detection (*p<0.05). The performance of the four types of features was similar, among which modularity was slightly better. These indicators highlight the dysfunction of information transmission and integration in the brain and network efficiency overdevelopment within the region, which can be significantly distinguished from healthy newborns. This provides a new and concise basis for clinical diagnosis, which could increase the general attention of clinicians.

Moreover, central node development in the left side was higher than that in the right side; specifically, there was preferential development of advanced language-related function of the newborn. The left side of the healthy newborn is usually 4.3% larger than the right side,^35^ which is consistent with our findings. Notably, infants with HIBD had missing regional central nodes responsible for language-related advanced cognitive functions, including channel 8 (left middle frontal gyrus), 15 (left upper frontal gyrus dorsal), and 30 (right central posterior gyrus). The central nodes are responsible for integrating information from each functional region to complete efficient resource allocation and operation; moreover, their absence could cause delayed cognitive function development in newborns. On the other hand, the sample size limits further discussion of distribution of the central nodes. Although the central nodes were not selected as features for classification in this study, their low level of development for HIBD infants makes them still worth collecting for verification, which may build a relationship between HIBD and functional networks.

Taken together, sustained brain damage could disrupt the development of key structural and functional networks, which leads to neurological development disorders in newborns. fNIRS-based analysis of the resting-state brain network could be applied to identify abnormal features regarding brain functional development in infants with HIBD, which contributes to the pathological understanding and clinical diagnosis of the disease. There are also some limitations and suggestions given based on our results. The small sample size restricts the statistical power to a certain extent. We have not made a more in-depth pathological analysis of HIBD. In future studies, fNIRS and clinical manifestations of HIBD will be combined to assess the association between the network of lesions and the core symptoms. Moreover, we expect to mine as many effective features as possible in a fixed threshold range for the needs of clinical diagnosis. The sparsity segment near 0.3 was a recommended threshold range and could be used as a reference for future research on infant brain functional connectivity.

## 5 Conclusion

This is the first study to conduct fNIRS analysis of the resting-state brain network for assessing brain function levels in children with HIBD.

By exploring brain network attributes, we observed significant between-group differences in various aspects, including functional connectivity intensity, node center, and information transmission between brain regions. These findings provide a theoretical basis for the clinical characterization of mental retardation, cerebral palsy, convulsion, and cognitive dysfunction in children. Further, according to specific network defects, the extracted sensitivity index based on small-world attributes, efficiency, modularity, and node degree could be effectively applied to identify patients, which indicates that fNIRS-based analysis of the resting-state brain network could be an exciting tool for assisting in the early clinical diagnosis of HIBD. Future studies should assess the utility of this technology for other types of neonatal brain injury.

## References

[1] Kurinczuk J. J. White-Koning M. Badawi N., “Epidemiology of neonatal encephalopathy and hypoxic-ischaemic encephalopathy,” Early Hum. Dev. 86(6), 329–338 (2010). EHDEDN 0378-3782 10.1016/j.earlhumdev.2010.05.010 20554402

[2] Douglas E. M. Weiss M. D., “Hypoxic ischemic encephalopathy: a review for the clinician,” JAMA Pediatr. 169(4), 397–403 (2015). 10.1001/jamapediatrics.2014.3269 25685948

[r3] Ferriero D. M., “Neonatal brain injury,” N. Engl. J. Med. 351(19), 1985–1995 (2004). NEJMAG 0028-4793 10.1056/NEJMra041996 15525724

[4] Hagberg H. David E. A. Groenendaal F., “Perinatal brain damage: the term infant,” Neurobiol. Dis. 92(Pt. A), 102–112 (2016). NUDIEM 0969-9961 10.1016/j.nbd.2015.09.011 26409031 PMC4915441

[5] Douglas E. M. Weiss M. D., “Biomarkers of hypoxic-ischemic encephalopathy in newborns,” Front. Neurol. 3, 144 (2012). 10.3389/fneur.2012.00144 23130015 PMC3486976

[6] Volpe J. J., “Neonatal encephalopathy: an inadequate term for hypoxic-ischemic encephalopathy,” Ann. Neurol. 72(2), 156–166 (2012). 10.1002/ana.23647 22926849

[7] Ferrari M. Quaresima V., “A brief review on the history of human functional near-infrared spectroscopy (fNIRS) development and fields of application,” NeuroImage 63, 921–935 (2012). NEIMEF 1053-8119 10.1016/j.neuroimage.2012.03.049 22510258

[8] Leff D. R. et al., “Assessment of the cerebral cortex during motor task behaviours in adults: a systematic review of functional near infrared spectroscopy (fNIRS) studies,” NeuroImage 54, 2922–2936 (2011). NEIMEF 1053-8119 10.1016/j.neuroimage.2010.10.058 21029781

[9] Fox M. D. Raichle M. E., “Spontaneous fluctuations in brain activity observed with functional magnetic resonance imaging,” Nat. Rev. Neurosci. 8(9), 700–711 (2007). NRNAAN 1471-003X 10.1038/nrn2201 17704812

[10] Den H. Pol H. E., “Exploring the brain network: a review on resting-state fMRI functional connectivity,” Eur. Neuropsychopharmacol. 20(8), 519–534 (2010). EURNE8 0924-977X 10.1016/j.euroneuro.2010.03.008 20471808

[11] Damoiseaux J. S. et al., “Consistent resting-state networks across healthy subjects,” Proc. Natl. Acad. Sci. U. S. A. 103(37), 13848–13853 (2006). 10.1073/pnas.0601417103 16945915 PMC1564249

[12] Raichle M. E. Snyder A. Z., “A default mode of brain function: a brief history of an evolving idea,” NeuroImage 37(4), 1083–1090 (2007). NEIMEF 1053-8119 10.1016/j.neuroimage.2007.02.041 17719799

[13] Friston K., “Beyond phrenology: what can neuroimaging tell us about distributed circuitry?” Annu. Rev. Neurosci. 25, 221–250 (2002). ARNSD5 0147-006X 10.1146/annurev.neuro.25.112701.142846 12052909

[14] Bullmore E. T. Sporns O., “Complex brain networks: graph theoretical analysis of structural and functional systems,” Nat. Rev. Neurosci. 10(3), 186–198 (2009). NRNAAN 1471-003X 10.1038/nrn2575 19190637

[15] Fransson P. et al., “Spontaneous brain activity in the newborn brain during natural sleep-an fMRI study in infants born at full term,” Pediatr. Res. 66(3), 301–305 (2009). PEREBL 0031-3998 10.1203/PDR.0b013e3181b1bd84 19531974

[16] Fransson P. et al., “Resting-state networks in the infant brain,” Proc. Natl. Acad. Sci. U. S. A. 104(39), 15531–15536 (2007). 10.1073/pnas.0704380104 17878310 PMC2000516

[17] Smyser C. D. et al., “Effects of white matter injury on resting state fMRI measures in prematurely born infants,” PLoS One 8(7), e68098 (2013). POLNCL 1932-6203 10.1371/journal.pone.0068098 23874510 PMC3706620

[18] Tusor N., “Diffusion tensor imaging and resting state functional connectivity as advanced imaging biomarkers of outcome in infants with hypoxic-ischaemic encephalopathy treated with hypothermia,” PhD Thesis, Imperial College London, London (2014).

[19] Homae F. et al., “Development of global cortical networks in early infancy,” J. Neurosci. 30(14), 4877–4882 (2010). JNRSDS 0270-6474 10.1523/JNEUROSCI.5618-09.2010 20371807 PMC6632781

[20] Makiko I. et al., “Functional connectivity of the cortex of term and preterm infants and infants with Down’s syndrome,” NeuroImage 85, 272–278 (2014). NEIMEF 1053-8119 10.1016/j.neuroimage.2013.04.080 23631984

[21] Lin C. Qi D. Haijing N., “The development of functional network organization in early childhood and early adolescence: a resting-state fNIRS study,” Dev. Cognit. Neurosci. 30, 223–235 (2018). 10.1016/j.dcn.2018.03.003 29631206 PMC6969083

[22] Kelsey C. M. et al., “Gut microbiota composition is associated with newborn functional brain connectivity and behavioral temperament,” Brain Behav. Immun. 91(2), 472–486 (2020). IUNIEH 1074-7613 10.1016/j.bbi.2020.11.003 33157257

[23] Wang J. Dong Q. Niu H., “The minimum resting-state fNIRS imaging duration for accurate and stable mapping of brain connectivity network in children,” Sci. Rep. 7(1), 6461 (2017). SRCEC3 2045-2322 10.1038/s41598-017-06340-7 28743886 PMC5527110

[24] Scholkmann F. et al., “How to detect andreduce movement artifacts in near-infrared imaging using moving standard deviation and spline interpolation,” Physiol. Meas. 31, 649–662 (2010). PMEAE3 0967-3334 10.1088/0967-3334/31/5/004 20308772

[r25] Sutoko S. et al., “Atypical dynamic-connectivity recruitment in attention-deficit/hyperactivity disorder children: an insight into task-based dynamic connectivity through an fNIRS study,” Front. Hum. Neurosci. 14, 3 (2020). 10.3389/fnhum.2020.00003 32082132 PMC7005005

[26] Jingping X. et al., “FC-NIRS: a functional connectivity analysis tool for near-infrared spectroscopy data,” Biomed. Res. Int. 2015, 1–11 (2015). 10.1155/2015/248724 PMC4619753 26539473

[27] Li Z. et al., “Assessment of cerebral oxygenation oscillations in subjects with hypertension,” Microvasc. Res. 88, 32–41 (2013). MIVRA6 0026-2862 10.1016/j.mvr.2013.04.003 23583904

[28] Van der Zee Z. P. et al., “Experimentally measured optical pathlengths for the adult head, calf and forearm and the head of the newborn infant as a function of inter optode spacing,” Adv. Exp. Med. Biol. 316, 143–153 (1992). AEMBAP 0065-2598 10.1007/978-1-4615-3404-4_17 1288074

[29] Achard S. Bullmore E., “Efficiency and cost of economical brain functional networks,” PLoS Comput. Biol. 3(2), e17 (2007). 10.1371/journal.pcbi.0030017 17274684 PMC1794324

[30] Bassett D. S. et al., “Hierarchical organization of human cortical networks in health and schizophrenia,” J. Neurosci. 28(37), 9239–9248 (2008). JNRSDS 0270-6474 10.1523/JNEUROSCI.1929-08.2008 18784304 PMC2878961

[31] Watts D. J. Strogatz S. H., “Collective dynamics of ‘small-world’ network,” Nature 393(6684), 440–442 (1998). 10.1038/30918 9623998

[32] Power S. Chau T., “Automatic single-trial classification of prefrontal hemodynamic activity in an individual with Duchenne muscular dystrophy,” Dev. Neurorehabil. 16, 67–72 (2013). 10.3109/17518423.2012.718293 23030232

[33] Xu B. et al., “Improving classification by feature discretization and optimization for fNIRS-based BCI,” Biomim. Biomater. Tissue Eng. 19, 1000119 (2014). 10.4172/1662-100X.1000119

[34] Elveda G. Nehal A. P. Stephanie L. M., “Altered functional network connectivity in preterm infants: antecedents of cognitive and motor impairments,” Brain Struct. Funct. 223, 3665–3680 (2018). 10.1007/s00429-018-1707-0 29992470 PMC6892636

[35] Fransson P. et al., “The functional architecture of the infant brain as revealed by resting-state fMRI,” Cereb. Cortex 21(1), 145–154 (2011). 53OPAV 1047-3211 10.1093/cercor/bhq071 20421249

